# Vet informatics and the future of drug discovery in veterinary medicine

**DOI:** 10.3389/fvets.2024.1494242

**Published:** 2024-11-27

**Authors:** Manos C. Vlasiou

**Affiliations:** Department of Veterinary Medicine, University of Nicosia School of Veterinary Medicine, Nicosia, Cyprus

**Keywords:** vet informatics, computer aided drug discovery, artificial intelligence, One Health, zoonotic diseases

## Introduction

Vet informatics—the application of data analytics, bioinformatics, and computational tools to manage and analyze biological and clinical data in veterinary science—is revolutionizing drug discovery and development. By harnessing vast datasets and advanced computational methods, vet informatics opens new avenues for identifying novel therapeutics, optimizing treatments, and repurposing existing drugs for animal health. However, its adoption faces challenges such as data standardization, privacy concerns, and economic barriers. This article evaluates the state of vet informatics, highlighting its strengths and limitations while proposing directions for its future integration into veterinary practice.

## Value and limitations of current methods

### Data integration and predictive analytics

Recent advancements in vet informatics have underscored the value of integrating diverse data types—from genomic and proteomic to clinical and environmental data—to accelerate drug discovery processes. These integrated datasets provide a comprehensive view of disease mechanisms, enabling the identification of novel drug targets with greater efficiency than traditional methods. Machine learning (ML) and artificial intelligence (AI) are particularly powerful for analyzing complex datasets, uncovering patterns, and predicting outcomes (1). For example, a study using ML models showed improved accuracy in predicting drug efficacy across different animal species, reducing the time and cost of preclinical trials (2).

However, data integration faces challenges. Lack of standardized data collection and sharing practices across veterinary institutions results in inconsistencies, lowering the reliability and reproducibility of findings (3). Additionally, integrating disparate datasets requires significant computational resources and expertise, which are often unavailable in smaller or resource-limited veterinary practices. Ensuring data security is also paramount, as the use of electronic health records (EHRs) raises concerns about data breaches and the misuse of sensitive information. To address these challenges, establishing standardized data protocols and ethical guidelines is essential for broad adoption (4).

### Applications in personalized veterinary medicine

Personalized veterinary medicine, where treatment plans are tailored based on an individual animal's genetic makeup, lifestyle, and environment, has made significant strides with the rise of vet informatics. This approach has shown promise in improving therapeutic outcomes and minimizing adverse reactions, similar to trends in human precision medicine. For example, incorporating genetic data into treatment strategies for canine cancer has optimized dosing regimens and reduced side effects (5). Furthermore, personalized approaches are proving beneficial in managing chronic diseases in livestock, such as metabolic disorders in dairy cows.

Despite these advancements, challenges remain. A key obstacle is the limited availability of comprehensive genetic and genomic databases for many veterinary species. While projects like the Canine Genome Project have improved our understanding of dogs, efforts to map the genomes of other species, such as felines, livestock, and exotic animals, are still in their infancy. Economic constraints also hinder the widespread adoption of personalized veterinary medicine (6). Genetic testing, bioinformatics analysis, and individualized treatment plans can be prohibitively expensive, particularly for small or underserved practices. Expanding public-private partnerships and reducing the cost of genetic testing could make personalized veterinary medicine more accessible (7).

### Drug repurposing opportunities

Vet informatics offers significant promise in drug repurposing, where existing medications are used for new veterinary applications. This approach is especially valuable given the high cost and regulatory barriers of developing new veterinary drugs. AI-based models, for instance, have been used to analyze existing pharmacological databases, identifying human drugs that could potentially treat diseases in animals (8). A notable example is the use of computational tools to repurpose antiviral drugs for treating respiratory conditions in livestock.

However, drug repurposing in veterinary medicine is constrained by several factors. The availability of pharmacological data for veterinary species is limited, and most existing databases focus on human medicine (9). Differences in drug metabolism between species mean that drugs effective in humans may not yield the same results in animals, necessitating rigorous preclinical and clinical trials. Collaborative efforts among researchers, regulators, and the pharmaceutical industry are essential for building comprehensive datasets and regulatory frameworks to accelerate drug repurposing (10).

## Critique of existing methods

While vet informatics offers many advantages, its utility is hampered by challenges. The lack of standardized data collection practices across institutions remains a primary concern. Inconsistent methodologies and data formats limit the reproducibility of results, complicating collaborations and data sharing. Furthermore, biases in data collection are a significant issue (11). For example, certain species or conditions may be overrepresented in veterinary datasets, skewing results and limiting the generalizability of findings. These biases can be mitigated by broadening data collection to include underrepresented species and conditions, along with developing robust ethical guidelines for data management (12).

## Economic considerations

The implementation of vet informatics requires significant investment in infrastructure and expertise. Small practices, especially those in rural or underserved areas, may find it challenging to adopt these technologies due to high costs (13). However, initiatives such as government subsidies, public-private partnerships, and reduced-cost technology solutions could help lower the barriers to adoption. For instance, partnerships between veterinary schools and tech companies could provide affordable access to AI tools and genetic testing services, enabling more widespread use of vet informatics (10).

## Future directions

To fully harness the potential of vet informatics in drug discovery, several key areas require further development. First, standardizing data collection and sharing protocols across veterinary institutions will improve research reliability and collaboration (14). Creating centralized repositories for veterinary genetic, clinical, and pharmacological data would facilitate research and clinical applications. Additionally, investment in education and training programs to enhance digital literacy and data analytics skills among veterinarians is crucial for bridging the gap between research and clinical practice (15).

Regulatory frameworks must evolve to address the ethical concerns of data privacy and security. Policymakers, veterinarians, and industry stakeholders must collaborate to develop guidelines that ensure the responsible use of veterinary health data. Addressing economic barriers through subsidized programs and partnerships will further promote the adoption of vet informatics, ensuring that its benefits are accessible to all veterinary practitioners, regardless of size or location.

## Conclusion

Vet informatics holds great potential to revolutionize drug discovery and development in veterinary medicine by providing new tools to identify novel therapeutics, optimize treatments, and repurpose existing drugs. However, realizing this potential requires addressing the challenges of data standardization, biases, economic feasibility, and privacy concerns. By investing in research, infrastructure, and education, the veterinary field can overcome these barriers and fully integrate informatics into advancing animal health.
